# Improved Contact Resistance by a Single Atomic Layer Tunneling Effect in WS_2_/MoTe_2_ Heterostructures

**DOI:** 10.1002/advs.202100102

**Published:** 2021-03-15

**Authors:** Jihoon Kim, A. Venkatesan, Hanul Kim, Yewon Kim, Dongmok Whang, Gil‐Ho Kim

**Affiliations:** ^1^ School of Electronic and Electrical Engineering Sungkyunkwan University (SKKU) Suwon 16419 Republic of Korea; ^2^ Samsung‐SKKU Graphene Centre Sungkyunkwan Advanced Institute of Nanotechnology (SAINT) Sungkyunkwan University (SKKU) Suwon 16419 Republic of Korea; ^3^ School of Advanced Materials Science and Engineering Sungkyunkwan University (SKKU) Suwon 16419 Republic of Korea

**Keywords:** contact resistance, monolayer, tunneling, vdW heterostructures, WS_2_/MoTe_2_

## Abstract

Manipulation of Ohmic contacts in 2D transition metal dichalcogenides for enhancing the transport properties and enabling its application as a practical device has been a long‐sought goal. In this study, n‐type tungsten disulfide (WS_2_) single atomic layer to improve the Ohmic contacts of the p‐type molybdenum ditelluride (MoTe_2_) material is covered. The Ohmic properties, based on the lowering of Schottky barrier height (SBH) owing to the tunneling barrier effect of the WS_2_ monolayer, are found to be unexpectedly excellent at room temperature and even at 100 K. The improved SBH and contact resistances are 3 meV and 1 MΩ µm, respectively. The reduction in SBH and contact resistance is confirmed with temperature‐dependent transport measurements. This study further demonstrates the selective carrier transport across the MoTe_2_ and WS_2_ layers by modulating the applied gate voltage. This WS_2_/MoTe_2_ heterostructure exhibits excellent gate control over the currents of both channels (n‐type and p‐type). The on/off ratios for both the electron and hole channels are calculated as 10^7^ and 10^6^, respectively, indicating good carrier type modulation by the electric field of the gate electrode. The Ohmic contact resistance using the tunneling of the atomic layer can be applied to heterojunction combinations of various materials.

## Graphical abstract

Innovative heterostructure consisting of a semiconducting monolayer WS_2_ tunneling layer and a few‐layer bottom MoTe_2_ is reported. By applying an appropriate gate voltage, n‐channel in WS_2_ monolayer and p‐channel in MoTe_2_ are accessed separately. Owing to single atomic thickness of WS_2_, the Schottky barrier and contact resistance on few‐layer MoTe_2_ are greatly reduced resulting in good Ohmic contact.

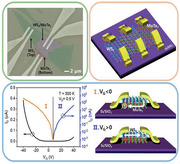

## 1 Introduction

2D transition metal dichalcogenides (TMDs) have been demonstrated as promising for future energy‐efficient nanoelectronics owing to their ultrathin body with enhanced electrostatic gating, carrier confinement, suitable bandgaps, passivated surfaces, good intrinsic carrier mobility, and mechanical flexibility.^[^ ^1^, ^2^, ^3^, ^4^, ^5^ ^]^ To fully explore the potential of TMDs for practical devices and circuit applications, the development of metal–semiconductor (MS) contact engineering and optimization is critical, which can maximize the device performance.^[^ ^6^, ^7^, ^8^ ^]^ In particular, for short‐channel devices, the contact condition between 2D semiconductors and metals is crucial in the entire carrier transport process.^[^ ^9^, ^10^ ^]^ To lower the Schottky barrier height (SBH) at the MS interface and to reduce the contact resistance (*R*_C_), several approaches have been proposed for TMD‐based nanoelectronic devices. Substitutional doping, surface charge transfer doping, isoelectronic alloying, hybridization, and phase engineering techniques have been used to reduce the contact barrier height.^[^ ^11^, ^12^, ^13^, ^14^ ^]^ On the metal side, the effect of the conventional work function engineering demonstrated that aluminum, scandium, and titanium contacts could provide low SBHs on molybdenum ditelluride (MoTe_2_).^[^ ^6^, ^15^, ^16^, ^17^ ^]^ Furthermore, novel contact architectures, such as 1D edge contact and high‐quality metal deposition conditions, such as ultrahigh vacuum can also result in low *R*_C_.^[^ ^18^ ^]^ Recently, a thin hexagonal boron nitride (h‐BN) layer was used as a spacer to isolate the metal electrode from the active channel material, thereby eliminating the interface states resulting in the Fermi level pinning, to improve the contacts.^[^ ^19^, ^20^ ^]^

Although metal/h‐BN contacts demonstrate high‐quality Ohmic contact, the wide range of possibilities of stacking MoTe_2_ with other 2D materials for creating an atomic‐scale heterostructure and superlattice can enable types of heterostructures that could not be implemented by MoTe_2_ devices previously, including negative differential resistance, ambipolar, tunneling, and photodevices.^[^ ^21^, ^22^, ^23^, ^24^ ^]^ Therefore, new alternatives are required for an insulating layer that not only improves electrical contacts but also provides further functionalities to existing devices.

Thus, in the fabrication techniques of smart and multifunctional integrated chips, a new paradigm has arisen. In this scenario, we have proposed and demonstrated the application of a semiconducting monolayer tungsten disulfide (WS_2_) both as a tunneling and active channel in a heterostructure device; thus, WS_2_ is used as an insulating layer, thereby enabling additional functionalities of the device. This study, based on the transport measurement depending on the temperature (100–300 K), shows that the single‐layer WS_2_ tunneling contact (WTC) on MoTe_2_ compared to direct contact with MoTe_2_ (MDC) has the effect of lowering the barrier between the metal–semiconductor, and thus the Ohmic contact resistance was improved. This single‐layer tunneling technology is an application device technology that can improve the Ohmic in a wide range of TMD materials.

## 2 Results and Discussion

**Figure 1:**
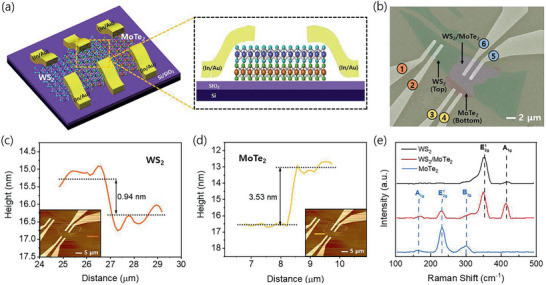
a) Schematic of the entire WS_2_/MoTe_2_ heterostructure device and a partial enlargement of the WTC contact. b) False‐color field emission scanning electron microscopy (FESEM) image of the WS_2_/MoTe_2_ heterostructure device with each contact numbered (WS_2_ direct contact (WDC), MDC, and WTC). c) Atomic force microscopy image and thickness of the monolayer WS_2_. d) Atomic force microscopy image and thickness of the few‐layer MoTe_2_. e) Raman spectrum of the monolayer WS_2_, few‐layer MoTe_2_, and WS_2_/MoTe_2_ heterostructures.

**Figure** **1**a,b shows the schematic and false‐color field emission scanning electron microscopy (FESEM) images of the dual‐channel WS_2_/MoTe_2_ heterostructure (DWM) field‐effect transistor (FET), wherein the monolayer WS_2_ is stacked on a few‐layer MoTe_2_. Following the dry transfer stacking of WS_2_ and MoTe_2_, metal electrodes (In/Au) were deposited such that the metal electrodes were only in contact with the top monolayer WS_2_. Figure 1e shows the Raman spectra of WS_2_ and MoTe_2_ layers obtained using a 532 nm laser at room temperature. In the WS_2_ Raman spectrum, the presence of signature peaks $E_{2g}^{1}$ (in‐plane) and A_1g_ (out‐of plane) at frequencies of ≈350 and ≈415 cm^−1^ confirms that the flake is WS_2_.^[^ ^25^ ^]^ The MoTe_2_ Raman spectrum comprises signature peaks A_1g_ (out‐of plane), $E_{2g}^{1}$ (in‐plane), and $B_{2g}^{1}$ modes at frequencies ≈168, ≈234, and ≈290 cm^−1^, respectively,^[^ ^26^ ^]^ which confirms that the flake is MoTe_2_. The Raman spectrum of the overlapped region comprises both WS_2_ and MoTe_2_ peaks, and a significant decrease in the intensity of both of these peaks can be observed to that of the individual layers. This can be attributed to quenching effect, where an additional recombination mechanism of the photogenerated electron–hole pairs at the heterointerface results in a significant drop in the spectrum intensity. The thicknesses of both WS_2_ and MoTe_2_ flakes were confirmed by atomic force microscopy (AFM), as shown in Figure 1c,d. The thickness of the WS_2_ flake was 0.94 nm, confirming that the WS_2_ flake was monolayer,^[^ ^27^ ^]^ whereas that of MoTe_2_ was 3.53 nm, confirming that the thickness of the MoTe_2_ flake had five layers.^[^ ^28^ ^]^

**Figure 2:**
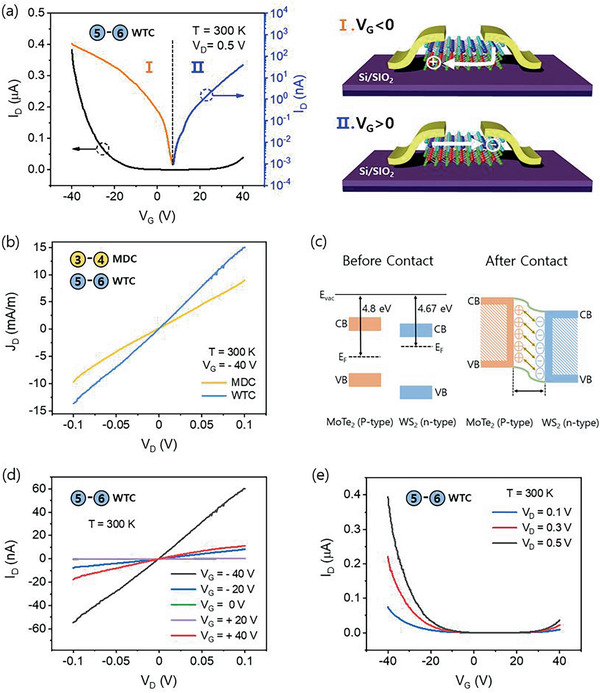
a) *I*_D_ *–V*_G_ characteristics of the DWM heterostructure FET and schematic of the current flowing through the WTC tunneling contacts at a drain voltage of 0.5 V (First diagram with positive and second diagram with negative gate voltage). b) *J*_D_ *–V*_D_ characteristics (at *V*_G_ = −40 V) of MoTe_2_ with WTC and MDC contacts with channel width (*W*) normalization. c) Energy band diagram of WS_2_ (n‐type) and MoTe_2_ (p‐type) before and after contact with a formation of depletion. d) *I*_D_ *–V*_D_ characteristics of the DWM heterostructure FET with WTC tunneling contacts at different gate voltages. e) *I*_D_ *–V*_G_ characteristics of the DWM heterostructure FET with WTC tunneling contacts at different drain voltages.

**Figure** **2**b shows the *J*_D_–*V*_D_ (at *V*_G_ = −40 V) characteristics of the MoTe_2_ with WTC and MDC contacts with W normalization. It can be confirmed from the near symmetric like *I*–*V* characteristics that both the WTC and the MDC contacts are of Ohmic. Furthermore, at a given source–drain voltage, the current in WTC contacts is higher than that of the MDC contact, confirming the superior quality of the WTC contact over the MDC contact. Figure 2a shows the *I*_D_ *–V*_G_ characteristics of the MoTe_2_ with WTC contact (Figures S2 and S4, Supporting Information, show the *I*_D_ *–V*_G_ characteristics of the MoTe_2_ and WS_2_ flakes with direct metal contacts (MDC and WDC), confirming that they were p‐type and n‐type materials, respectively). It is apparent from the *I*_D_ *–V*_G_ characteristics that the DWM heterostructure with WTC contact exhibits ambipolar characteristics with on/off ratios 10^7^ and 10^6^ for the positive and negative gate voltages, respectively. The first schematic in Figure 2a shows the *I*_D_ *–V*_G_ characteristics for the positive gate voltage with direct contact, and the second schematic shows *I*_D_ *–V*_G_ characteristics for the negative gate voltage with tunneling. The *I*_D_ *–V*_G_ characteristics of the WS_2_ with WDC contact (Figure S4, Supporting Information) confirmed that it is an n‐type material. Therefore, the ambipolar characteristics of WTC contact with dominant p‐type transport can be attributed to the tunneling nature of the contacts. The DWM FET with WTC contact showed a minimum conductivity at *V*_G_ ≈ 10 V, which could be attributed to the formation of a depletion region at the interface owing to the charge transfer between the WS_2_ and MoTe_2_ during the heterostructure formation. However, the conductivity of device increased when the gate voltage was further increased in both the negative and positive directions. The change in conductivity of the DWM was reflected in their *I*_D_ *–V*_G_ characteristics, where a minimum current was observed when the gate voltage was near the minimum conductivity voltage (*V*_G_ ≈ 10 V) than that of the higher gate voltage ranges (*V*_G_ = −30 and +30 V).

Figure 2d shows the *I*_D_ *–V*_D_ characteristics of the DWM heterostructure with WTC contact at different gate voltages. They exhibited linear characteristics at all gate voltages (at a positive gate voltage, the active channel was WS_2_, whereas at a negative gate voltage, the active channel was MoTe_2_), indicating the formation of good Ohmic contacts.^[^ ^29^ ^]^ Similarly, the DWM heterostructure with WTC tunneling contact exhibited ambipolar characteristics at different drain voltages. The observation of ambipolar transport at different drain voltages and the linear *I*_D_ *–V*_D_ characteristics (at different gate operating voltages) can be explained using Figure 2c. As discussed in the previous section, the transfer of WS_2_ over the MoTe_2_ layers results in a charge transfer and formation of a depletion layer at the heterointerface.^[^ ^30^ ^]^ This results in an apparent charge in neutrality in the heterostructure, which can also be seen in the *I*_D_ *–V*_G_ characteristics (Figure 2e).^[^ ^31^, ^32^ ^]^ As the gate sweeps to the positive regime, the downward band bending under the influence of the positive gate electric field results in an electron accumulation in the WS_2_ and a further depletion of charge carriers in the MoTe_2_. This results in an exponential increase in the current flowing through the WS_2_ channel through the metal electrodes, as can be seen in the *I*_D_ *–V*_G_ characteristics. In the negative gate regime, reverse band bending occurs owing to the negative gate electric field which results in the accumulation of holes in the MoTe_2_ and the formation of a current channel. As there is no direct contact between the MoTe_2_ and metal electrodes, the only possible way for carrier transport is tunneling through the intermediate WS_2_ layers from the metal electrodes to the underlying MoTe_2_. This explanation is further validated by the fact that in the negative gate regime, WS_2_ is fully depleted of carriers and acts like a dielectric tunneling layer, thus, forming a metal–semiconductor–insulator (MIS) contact to the underlying MoTe_2_ active channel.

The incorporation of top layer in the DWM heterostructure device results in dual‐channel transport without any metal contact engineering or doping processes. To further understand the operating behavior of the DWM device, we carried out transport measurements at different temperatures. Henceforth, for convenience, we refer to a DWM dual‐channel device as MoTe_2_ with WTC contacts.

**Figure 3:**
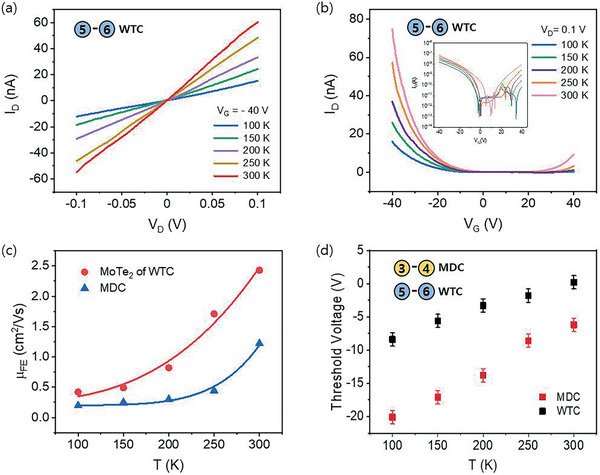
a) *I*_D_ *–V*_D_ characteristics of the DWM heterostructure with WTC contact at different temperatures in the range of 100–300 K at the gate voltage of −40 V. b) Drain‐dependent (0.1–0.5 V) *I*_D_ *–V*_G_ characteristics of the DWM heterostructure with WTC contact at different temperatures in the range of 100–300 K with gate voltages from −40 to +40 V. c) Variation in the mobility of WTC and MDC contacts with respect to temperature in the range of 100–300 K. d) Variation of threshold voltage of the WTC and MDC contacts with respect to temperature in the range of 100–300 K.

The performance of MoTe_2_ FET with WTC (**Figure** **3**a,b) and MDC (Figure S2c,d, Supporting Information) contacts is assessed by carrying out temperature‐dependent transport measurements in the temperature range of 100–300 K. The field effect mobility (μ_FE_), defined as (*L*/*W*) (1/*C*_ox_) (1/*V*_D_) (∂*I*_D_/∂*V*_G_), is calculated from the *I*_D_ *–V*_G_ characteristics as a function of temperature *T* and shown in Figure 3c. Here, *L* is the channel length, *W* is the average channel width, and *C*_OX_ is the capacitance of 285‐nm‐thick SiO_2_. The dependence of mobility on temperature *T* can be described by the power law μ_FE_ ≈ *T^*γ*^* and can directly indicate the difference in the carrier transport mechanism. It is interesting to see that the mobility of both WTC and MDC contacts shows similar patterns and strong and consistent dependence on temperature. The value of the exponent *γ* is found to be 2.50 and 2.94 for WTC and MDC contacts, respectively (Figure 3c). The temperature‐dependent threshold voltages (*V*_th_) were plotted for both contacts as shown in Figure 3d. As the temperature was increased in the range of temperature (100–300 K), in both cases, we observed positive shift in *V*_th_. Furthermore, we observed that the shift in *V*_th_ was less dependent on gate voltage in the case of WTC contact in comparison to MDC.

According to the thermionic emission theory, the current in the device can be described as follows^[^ ^20^ ^]^

(1) $$I\;=\;AA^{*}T^{3/2}\exp\left(-\frac{e\emptyset_{B}}{k_{B}T}\right)\exp\left(\frac{eV_{B}}{k_{B}T}-1\right)$$

where *I* is the current, *A* is the area of the device, A* is the modified Richardson constant, ∅_B_ is the SBH, *V*_D_ is the drain voltage, *e* is the electron charge, *k*_B_ is the Boltzmann constant, and *T* is the temperature in K. SBH, ∅_B_, obtained using this equation is not equivalent to that of the conventional MS junctions. According to the expression of *I*–*V* characteristics (Equation (1)), the insulator is not considered when the SBH ∅_B_ is determined; thus, the effective SBH ∅_B_ obtained from Equation (1) is the overall representation of the electrical behavior of the device. When the gate bias is above the flat band voltage, *V*_FB_, both components contribute to the current flow during the temperature‐dependent measurements. The contribution from the tunneling current becomes negligible only when the gate voltage is less than or equal to *V*_FB_.^[^ ^33^ ^]^

**Figure 4:**
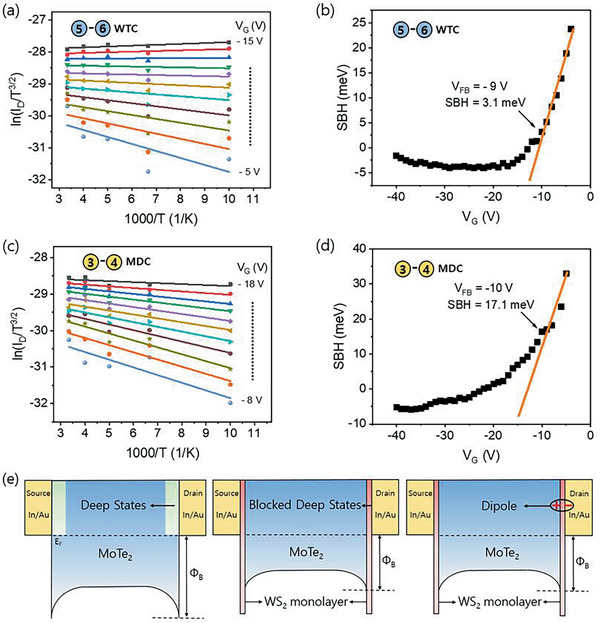
a) Arrhenius plot of the WTC contact as a function of the gate voltage (from *V*_G_ = −5 V to *V*_G_ = −15 V) for observing the change in slope at temperatures in the range of 100–300 K. b) SBH of the WTC contact as a function of gate voltage. c) Arrhenius plot of the MDC contact as a function of the gate voltage (from *V*_G_ = −8 to −18 V) for observing the change in slope at temperatures in the range of 100–300 K. d) SBH of the MDC contact as a function of gate voltage. e) Band diagram of the MoTe_2_ with direct indium contact and the progress of blocking deep states and dipole formation owing to the WS_2_ tunneling.

**Figure 5:**
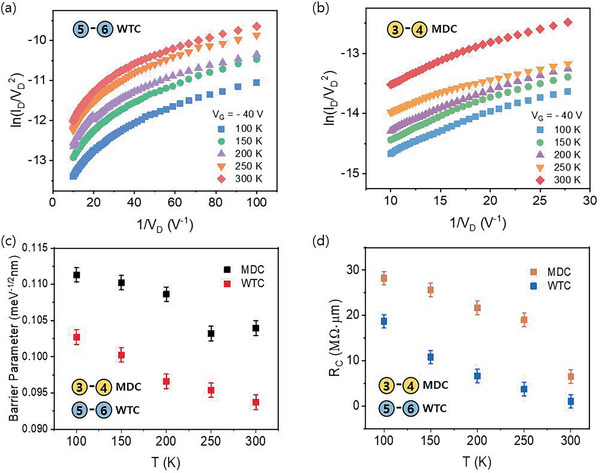
a) ln$\left(I_{D}/V_{D}^{2}\right)$ as a function of 1/*V*_D_ at *V*_G_ = −40 V for all temperatures in the range of 100–300 K for the WTC contact. b) ln$\left(I_{D}/V_{D}^{2}\right)$ as a function of 1/*V*_D_ at *V*_G_ = −40 V for all temperatures in the range of 100–300 K for MDC contact. c) Temperature‐dependent DT barrier obtained from the ln$\left(I_{D}/V_{D}^{2}\right)$ versus 1/*V*_D_ plot at *V*_G_ = −40 V for all temperatures in the range of 100–300 K for both the WTC and the MDC contacts. d) Contact resistance of the MDC and WTC contacts calculated using the *Y*‐function method.

To obtain an accurate ∅_B_ based on the thermionic emission theory and diode (Equation (1)), the SBH ∅_B_ needs to be determined at the flat band voltage condition (*V*_GS_ = *V*_FB_). To obtain ∅_B_ using Equation (1), we plotted various values of ln  (*I*_D_/*T* ^3/2^) at a fixed drain bias of 0.1 V for various gate biases as an Arrhenius plot. The slope of these lines directly provides the effective SBH ∅_B_ (in eV) for the corresponding gate bias, *V*_GS_. For comparison, Arrhenius graphs are plotted for WTC and MDC contacts for all gate voltages, and the slope (i.e., the effective ∅_B_) is plotted as a function of the gate voltage, as shown in **Figure** **4**a,c. The Schottky barrier, for *V*_GS_ less than or equal to *V*_FB_, obtained from Figure 4b,d linearly corresponds to *V*_GS_, as shown in Figure 4b,d. However, as *V*_GS_ increases above *V*_FB_, the tunneling current component becomes relevant, and the plot deviates from its linear relation. Hence, the accurate ∅_B_ value obtained at flat band voltage conditions for devices with the monolayer WS_2_ was 3 meV. Compared to this, the ∅_B_ value of the MoTe_2_ without any monolayer WS_2_ was 18 meV, which is six times higher than that of the tunneling contacts. Furthermore, to understand the transport characteristics of the MoTe_2_ with the WTC and MDC contacts at low temperature in the range of 100–250 K, we used the Fowler–Nordheim tunneling (F–N) and direct tunneling (DT) models. The FNT and DT tunneling mechanisms are described by the following equations^[^ ^34^, ^35^ ^]^

(2) $$\ln\left(\frac{I}{V^{2}}\right)\;\propto \;-\frac{1}{V}\left(\frac{8\pi d\sqrt{2m^{*}\emptyset_{B0}^{3}}}{3hq}\right)\;$$

(3) $$\ln\left(\frac{I}{V^{2}}\right)\;\propto \;\ln\left(\frac{1}{V}\right)-\;\frac{4\pi d\sqrt{2m^{*}\emptyset_{B0}}}{h}$$

where ∅_B0_ is the tunneling barrier height, *m* is the free electron mass, m* (0.46 m) is the effective mass of the electrons in the MoTe_2_ flake, *q* is the electron charge, *h* is Planck's constant, and *d* is the width of the interface barrier (the thickness of the MoTe_2_ for the MDC contact and 0.7 nm for the WTC contact). In addition, when ln (*I*/*V* ^2^) is plotted as a function of 1/V, all curves (at all temperatures) exhibit logarithmic dependence, and 1/*V* indicates that the dominant mechanism of charge carrier injection is because of DT (**Figure** **5**a,b). We could not observe signs of F–N tunneling in both the WTC and the MDC contact devices, as usually observed in several studies. The low voltage bias of the device can be attributed to the absence of the F–N tunneling (because the F–N tunneling is a dominant phenomenon only at higher voltage bias regions).^[^ ^34^ ^]^

The tunneling barrier height was obtained from the graphs 1/*V* versus $\ln\;(I_{D}/V_{D}^{2})$ plotted for both the WTC and MDC contacts shown in Figure 5c. For the MDC contact, the tunneling barrier parameter varied in the range of 0.24–0.16 meV^−1/2^ nm when the temperature was increased in the range of 100–300 K. For the WTC contacts, the tunneling barrier height varied in the range of 0.24–0.16 meV^−1/2^ nm when the temperature was decreased in the range of 300–100 K. As can be clearly seen from the above result, the tunneling barrier heights in the WTC contacts are well below the MDC contacts at all temperatures. The decrease in the tunneling barrier heights in the WTC contacts compared to those of the MDC contacts can be attributed to the insertion of the WS_2_, which prevents the formation of an interfacial barrier layer at the contact.

The superior performance of the WTC contact, including the increased current, and low Schottky barrier and tunneling barrier parameter can be interpreted by lowering of the barrier and the quantum tunneling induced by the monolayer WS_2_ at the MoTe_2_ interface (Figure 4e). Specifically, the conventional MS contact between the In and MoTe_2_ forms a Schottky barrier with an interacting interface, and the surface potential, defined as the difference between the metal and the semiconductor work functions, is completely located at the semiconductor side. In contrast, the MIS contact has a monolayer WS_2_ between In and MoTe_2_ as shown in Figure 4e. These additional barriers with a total thickness of ≈1 nm are still sufficiently thin to allow quantum tunneling to occur with a high tunneling probability; however, at the same time, they are sufficiently thick to share the original surface potential, as well as the band bending on the MoTe_2_ side and thus the effective SBH.

For an MIS structure, the contact resistance is mainly determined by the Schottky barrier and tunneling resistance. Inserting an insulating layer at the contact reduces the SBH but increases the tunneling resistance. Therefore, to understand the effect of insertion of the WS_2_ on the contact resistance, the contact resistances owing to the WTC and MDC contacts were evaluated using the *Y*‐function (Ghibaudo) method.^[^ ^36^, ^37^ ^]^ Using this method, the contact resistance can be obtained accurately from two‐terminal electrical measurements without the need of any special measuring techniques such as the transmission length method or four probe methods. First, the low‐field mobility (*μ*_0_) is obtained using the following equation^[^ ^36^ ^]^

(4) $$Y\;=\;\frac{I_{D}}{\sqrt{g_{m}}}=\;\sqrt{\frac{W}{L}C_{\mathrm{ox}}\mu_{0}V_{D}}\times \;\left(V_{G}-V_{T}\right)$$

where *I*_D_ is the drain current, *g*_m_ is the transconductance defined as d*I*_D_/d*V*_G_, *W* is the channel width, *L* is the channel length, *C*_ox_ is the gate capacitance, *μ*_0_ is the low‐field mobility which is shown in Equation (4), *V*_D_ is the applied drain bias, and *V*_G_ and *V*_T_ are the applied gate and threshold voltages, respectively.

Next, the mobility attenuation factor (*θ*) resulting from the contact resistance is calculated by using the following expression

(5) $$g_{m}=\;\frac{W}{L}C_{\mathrm{ox}}\frac{\mu_{0}}{{\left(1+\theta \left(V_{G}+V_{T}\right)\right)}^{2}}$$

Finally, the contact resistance is calculated using the relation *θ*  = *G*_m_  × *R*_C_, where *G*_m_ =  (*W*/*L*)*μ*_0_ *C*_ox_ is the transconductance parameter and *R*_C_ is the contact resistance.

Figure 5d shows the contact resistance of the WTC and MDC contacts. The contact resistances of both contacts clearly depend on the temperature. The WTC contact resistance increased from 1.0 to 18.7 MΩ µm when the temperature was decreased in the range of 300–100 K. A similar trend was observed for the MDC contacts, where the contact resistance increased from 6.5 MΩ µm at 300 K to 28.2 MΩ µm at 100 K. In the case of the WTC contacts, the contact resistances are lower at all temperatures than those of the MDC contacts. Particularly, at 100 K, the improvement in the contact resistance in the WTC contact is less than an order of magnitude. This can be attributed to the reduction in the SBH at the contacts, as discussed earlier.

In conclusion, we demonstrated innovative heterostructure consisting of single atomic thickness WS_2_ tunneling layer and few‐layer bottom MoTe_2_. The monolayer WS_2_ acted as an Ohmic contact tunneling layer on MoTe_2_ as well as channel material. Owing to single atomic thickness of WS_2_, the Schottky barrier on few‐layer MoTe_2_ was greatly reduced with decrease in tunneling resistance. The WS_2_ tunneling contacts on MoTe_2_ showed much improved Schottky barrier and contact resistance values of 3 meV and 1 MΩ µm, respectively. Furthermore, we also demonstrated that, in the WS_2_/MoTe_2_ heterostructure, by applying an appropriate gate voltage n‐channel in monolayer WS_2_ and p‐channel in MoTe_2_ can be accessed separately. The on/off ratios for electron and hole channels were calculated as 10^7^ and 10^6^, respectively, confirming the excellent carrier modulation by the gate electrode. By carrying out temperature (100–300 K) dependent transport measurements, we further confirmed the good quality of Ohmic contacts. Our work shows innovative contact method where monolayer WS_2_ acts as a tunneling layer as well as channel material. This device structure can open up a new avenue for 2D materials for future electronic devices.

## 3 Experimental Section

##### Device Fabrication

The WS_2_/MoTe_2_ heterostructure was fabricated by dry transfer method. First, a few‐layer MoTe_2_ flake was exfoliated from a bulk crystal (2D Semiconductors Inc.) on a precleaned and highly p‐doped Si substrate covered by a 300 nm thick SiO_2_ layer, using a conventional Scotch tape method. Then, the suitable flakes were identified by optical microscopy. To obtain monolayer WS_2_, for the stacking on the selected MoTe_2_ flake, WS_2_ pristine was exfoliated on a Si substrate covered by a 300 nm thick SiO_2_ layer. A suitable monolayer WS_2_ was selected by optical microscopy and stacked on the target MoTe_2_ flakes by dry transfer method. During the dry transfer, the pickup of the selected monolayer WS_2_ flake was carried out using polydimethylsiloxane (PDMS) base substrate covered by a polycarbonate (PC) sacrificial layer. Then, the monolayer WS_2_ flake was deterministically released on the targeted MoTe_2_. The residual PC was removed by immersing the sample in chloroform for 20 min followed by a nitrogen blow. After the transfer the sample was vacuum annealed at 150 °C for 1 h. Electrodes were patterned using e‐beam lithography followed by metal deposition In (10 nm)/Au (40 nm) in an electron‐beam deposition chamber and then annealed at 150 °C for 3 h in the presence of Ar flow (1000 SCCM).

##### Device Characterizations

Raman spectroscopy was performed to characterize the flakes using a 532 nm laser under ambient conditions. The thicknesses of the WS_2_ and MoTe_2_ flakes were determined using AFM. The electrical characterization of the device was performed under vacuum in a dark environment using a Keithley 4200‐SCS parameter analyzer.

## Conflict of Interest

The authors declare no conflict of interest.

## Author Contributions

J.K. and A.V. contributed equally to this work. A.V. and J.K. performed the fabrication, temperature‐dependent electrical measurements, data analysis, and manuscript writing. H.K. performed the device characterizations. D.W. contributed in modifying the manuscript. G.‐H.K. conceived the projects and supervised from the fabrication to data analysis and manuscript writing.

## Supporting information

Supporting Information

## Data Availability

Research data are not shared.
