# Supplementary material for: Improved Contact Resistance by a Single Atomic Layer Tunneling Effect in WS2/MoTe2 Heterostructures
Source: Adv Sci (Weinh). 2021 Mar 15;8(11):2100102. doi: 10.1002/advs.202100102 (PMC8188188; doi:10.1002/advs.202100102)
Supplement: Supplementary file 1 — Supporting Information [file ADVS-8-2100102-s001.pdf]

© 2021 Wiley-VCH GmbH

ADVANCED SCIENCE
Open Access

Supporting Information

for *Adv. Sci.*, DOI: 10.1002/advs.202100102

Improved Contact Resistance by a Single Atomic Layer Tunneling Effect in WS_2/$MoTe_2$ Heterostructures

*Jihoon Kim, A. Venkatesan, Hanul Kim, Yewon Kim, Dongmok Whang and Gil-Ho Kim**

Improved Contact Resistance by a Single Atomic Layer Tunneling Effect in $WS_2/MoTe_2$ Heterostructures

Jihoon Kim, A. Venkatesan, Hanul Kim, Yewon Kim, Dongmok Whang and Gil-Ho Kim[*]

-Supporting Information-

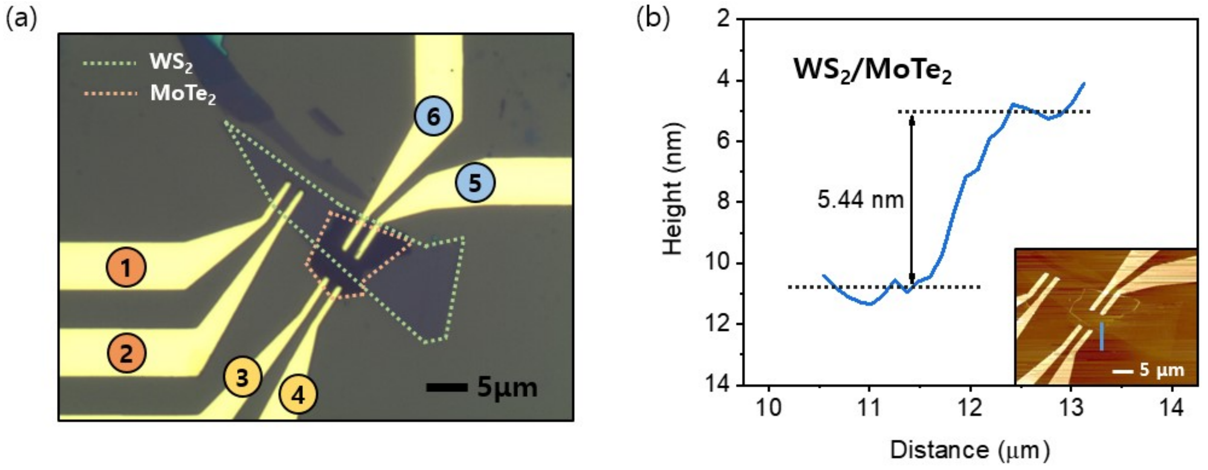

Figure S1. a) Optical image of the $WS_2/MoTe_2$ heterostructure device with each contact numbered. b) AFM measurement of $WS_2/MoTe_2$ which proves the thickness of the $WS_2/MoTe_2$ structure.

Figure S1a shows the optical microscopy image of the $WS_2/MoTe_2$ heterostructure. Figure S1b is the AFM micrograph of the $WS_2/MoTe_2$. The thickness of the heterostructure is found to be ~5.44 nm and it is equivalent to the thickness of both WS_2 and $MoTe_2$ layers.

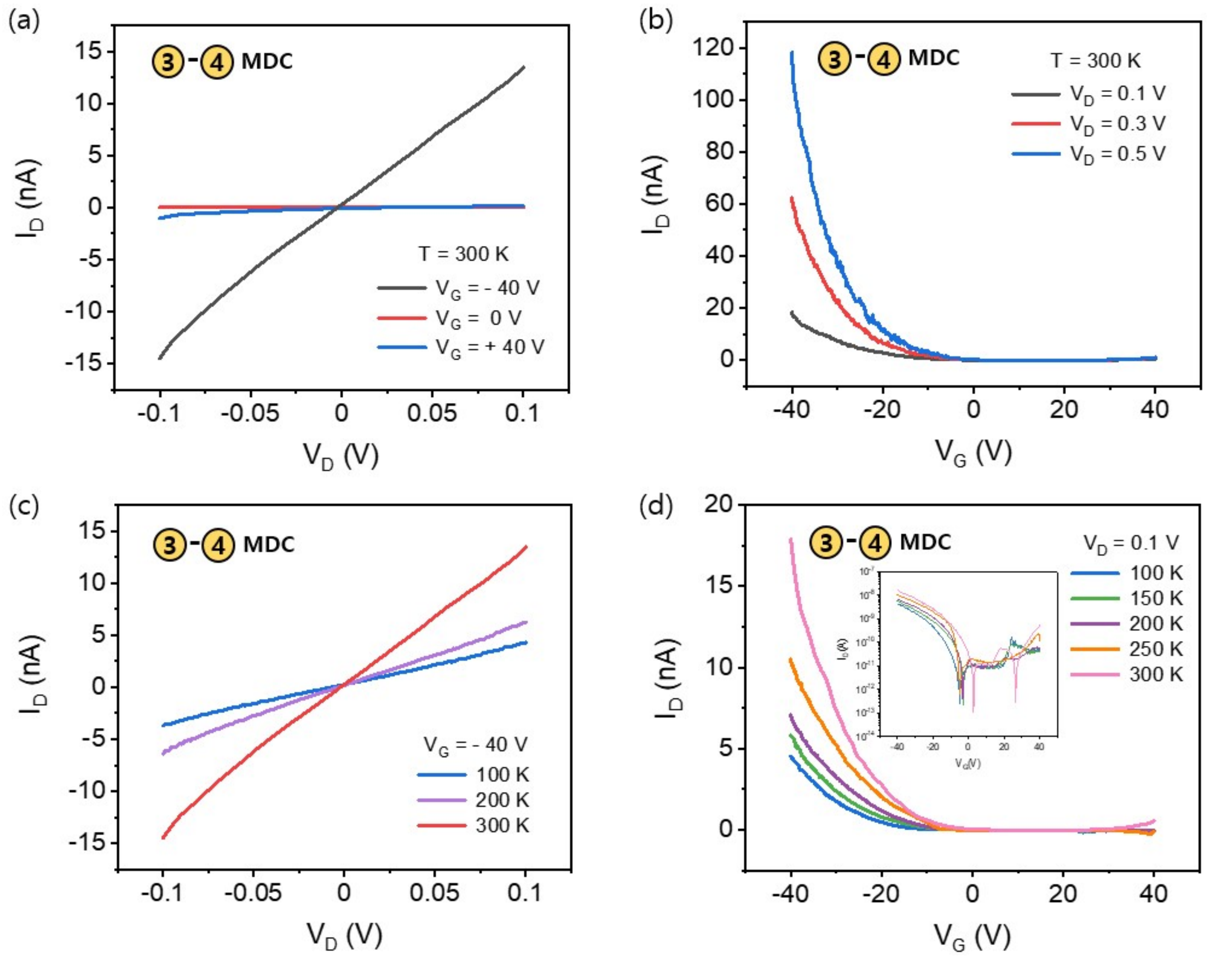

Figure S2. a) I_D-V_D characteristics of MDC direct contacts at different gate voltages. b) I_D-V_G characteristics of MDC direct contacts at different drain voltages. c) I_D-V_D characteristics of MDC direct contacts at different temperatures in the range of 100-300 K at the gate voltage −40 V. d) I_D-V_G characteristics of MDC contact at different temperatures in the range of 100-300 K with gate voltage from −40 to +40 V at the drain voltage 0.1 V.

Figure S2a is the I_D-V_D characteristics at different gate voltages. As the gate voltage was swept from −40 to +40 V, the current was decreasing gradually confirming that the channel is dominated by p-type charge carriers. In the case of I_D-V_G characteristics, a similar trend was observed for all V_D values, that is, as the gate voltage became more negative the drain current

increased. We also carried out the temperature dependent measurements in the range of 100-300 K. As the temperature was reduced in the range of 300-100 K, in both I_D-V_D and I_D-V_G characteristics, the current was reduced drastically indicating that decrease in the charge carrier due to a reduction in the thermal energy.

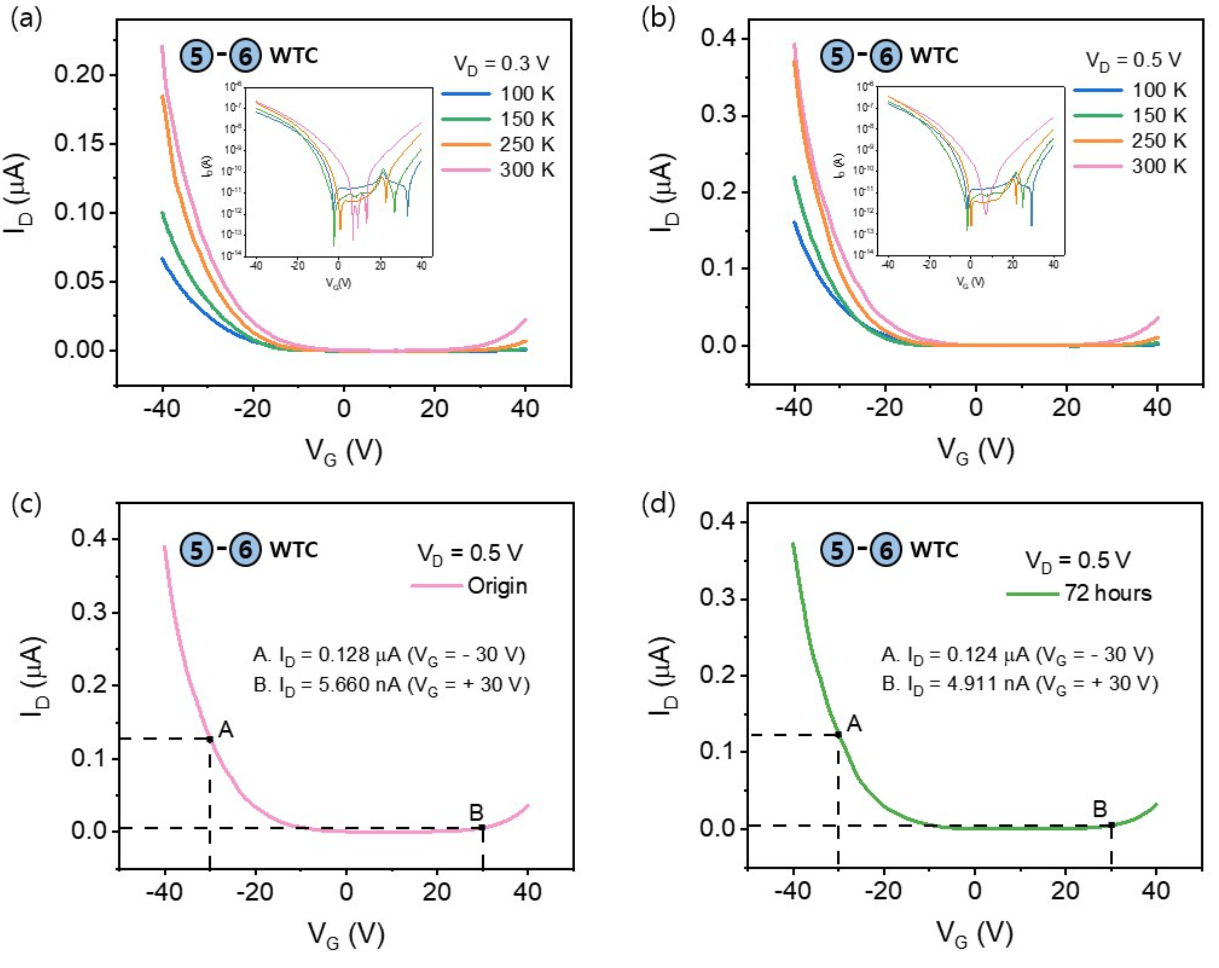

Figure S3. a) I_D-V_G characteristics of WTC contact at different temperatures in the range of 100-300 K with gate voltage from −40 to +40 V at the drain voltage 0.3 V, and b) 0.5 V. c) Original I_D-V_G curve of WTC contact with a specific point A (V_G = -30 V), and B (V_G = +30 V) for calculating the device stability at room temperature. d) I_D-V_G curve of WTC contact measured

after 72 hours with a specific point A (V_G = -30 V), and B (V_G = +30 V) for calculating the device stability at room temperature.

Figure S3a and S3b is the I_D-V_G characteristics at different temperature with drain voltage 0.3 V and 0.5 V. Tunneling current decreased as the temperature reduced in the range of 100-300 K at the drain voltage 0.3 V and 0.5 V.

For the viability of practical device applications, we also carried out the device stability studies. After the completion of temperature dependent transport measurements, we left our sample in ambient condition for 3 days (72 hours). After 3 days, we carried out similar transport measurements on our sample. Figure S3c and S3d show the transfer characteristics of the sample before the temperature dependent transport measurements and after 3 days measured at room temperature. At a given back gate voltage (Vg = - 30 V), we observed a slight decrease in the drain current (I_D) from 0.128 ± 0.001μA to 0.124 ± 0.001μA. This small decrease (~3%) in the drain current even after the temperature dependent measurements and leaving the sample in ambient condition for 3 days confirming the stability of our device.

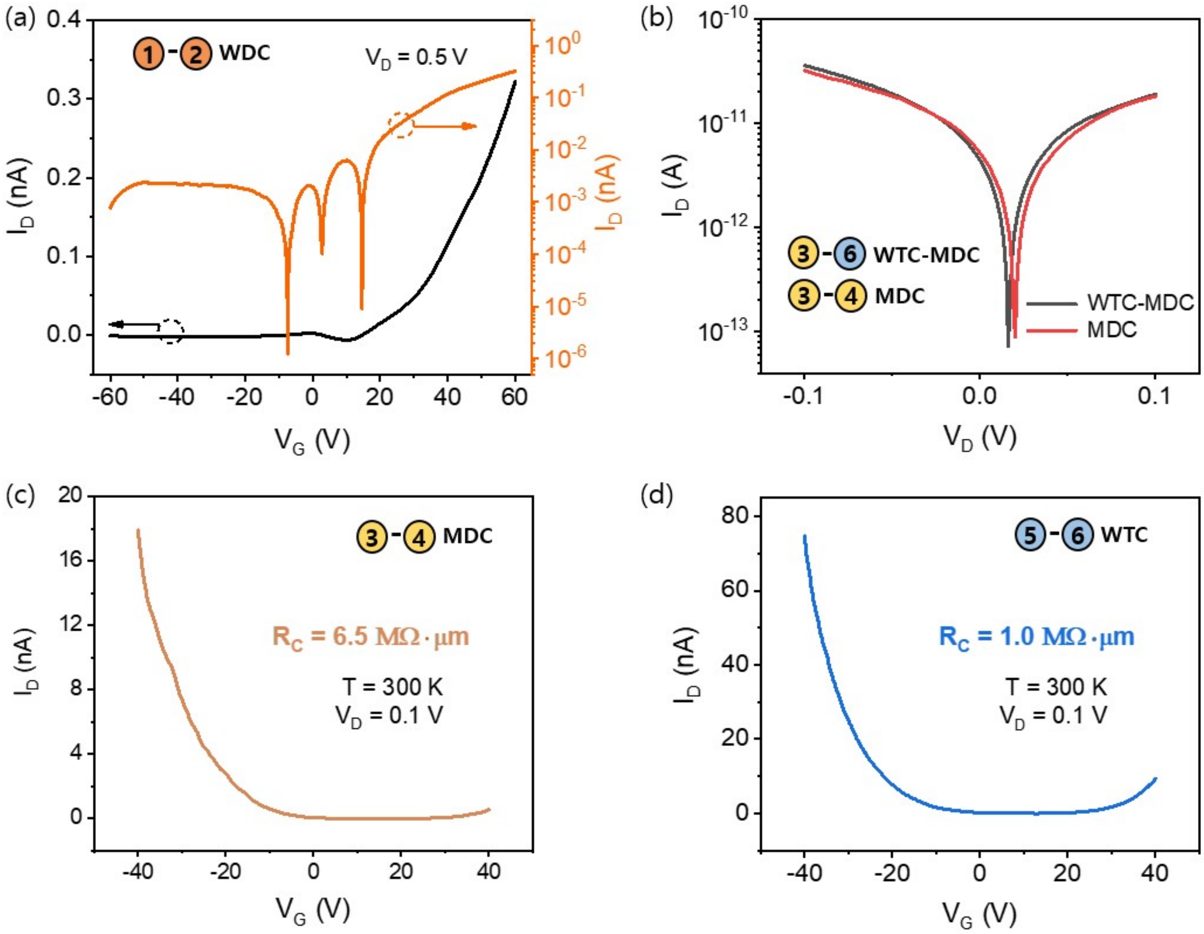

Figure S4. a) I_D-V_G characteristics of WDC contacts at the drain voltage 0.5 V. b) Comparison of the MDC contact and the WTC-MDC contact (one contact is MDC and the other is WTC contact). c) I_D-V_G curve of the MDC contact with a contact resistance calculated by the Y-function method at room temperature. d) I_D-V_G curve of the WTC contact with a contact resistance calculated by the Y-function method at room temperature.

We observed the I_D-V_G curve of WDC contact. Figure S4a is the I_D-V_G characteristics at different gate voltages from −60 V to +60 V. It shows n-type property which only shows electrical signals at positive gate voltage.

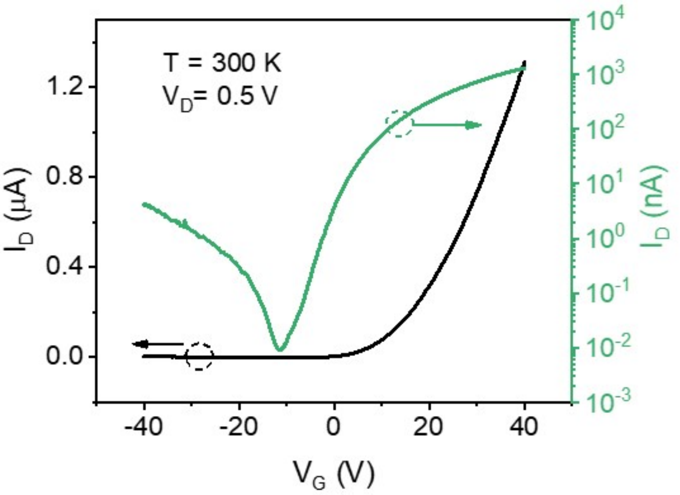

Figure S5. I_D-V_G characteristics of tunneling contacts with few layer WS_2 at the drain voltage 0.5 V which shows inferior tunneling properties compared with monolayer.

In order to confirm the tunneling current according to the thickness, a WS_2/$MoTe_2$ heterostructure device with a few-layer WS_2 was fabricated and compared with a monolayer. From the I_D-V_G characteristics in Figure S5, we can infer that the current in the few-layer WS_2 channel (at positive gate voltages) was more dominant in comparison to tunneling (at negative gate voltage) current indicating that the few-layer WS_2 is not suitable for forming good ohmic contacts. This further confirms that monolayer WS_2 is the optimized tunneling layer for excellent tunneling contacts.

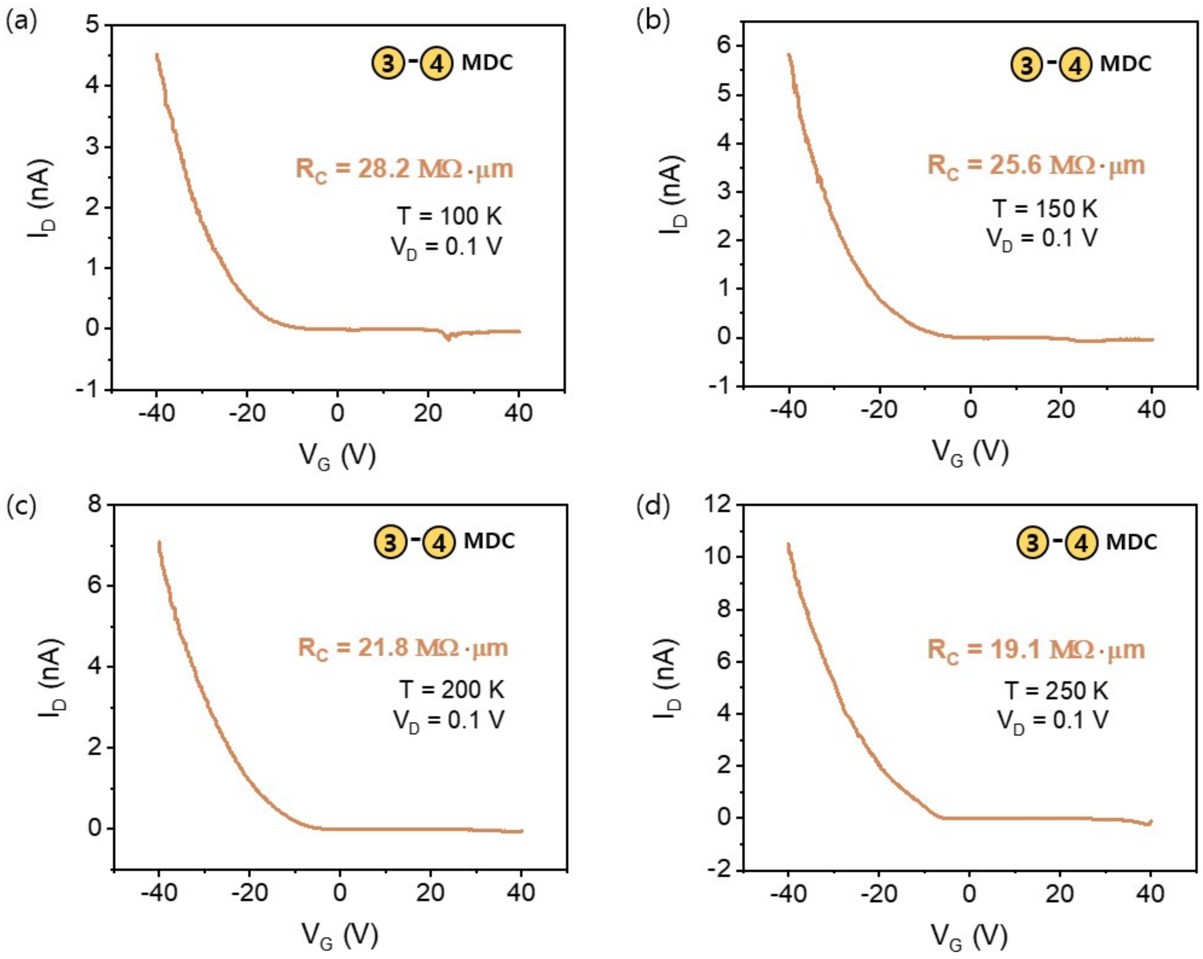

Figure S6. a) I_D-V_G curve of the MDC contact with a contact resistance calculated by the Y-function method at the drain voltage 0.1 V at 100 K, b) 150 K, c) 200 K, and d) 250 K.

To compare the MDC and WTC contacts, the contact resistances were calculated using the Y-function method [1-3] at room temperature (Figure S4c and S4d) and at low temperature in the range of 100-250 K (Figure S6 and S7). As observed in the Schottky barrier calculation, the contact resistance also decreased on the WTC contacts.

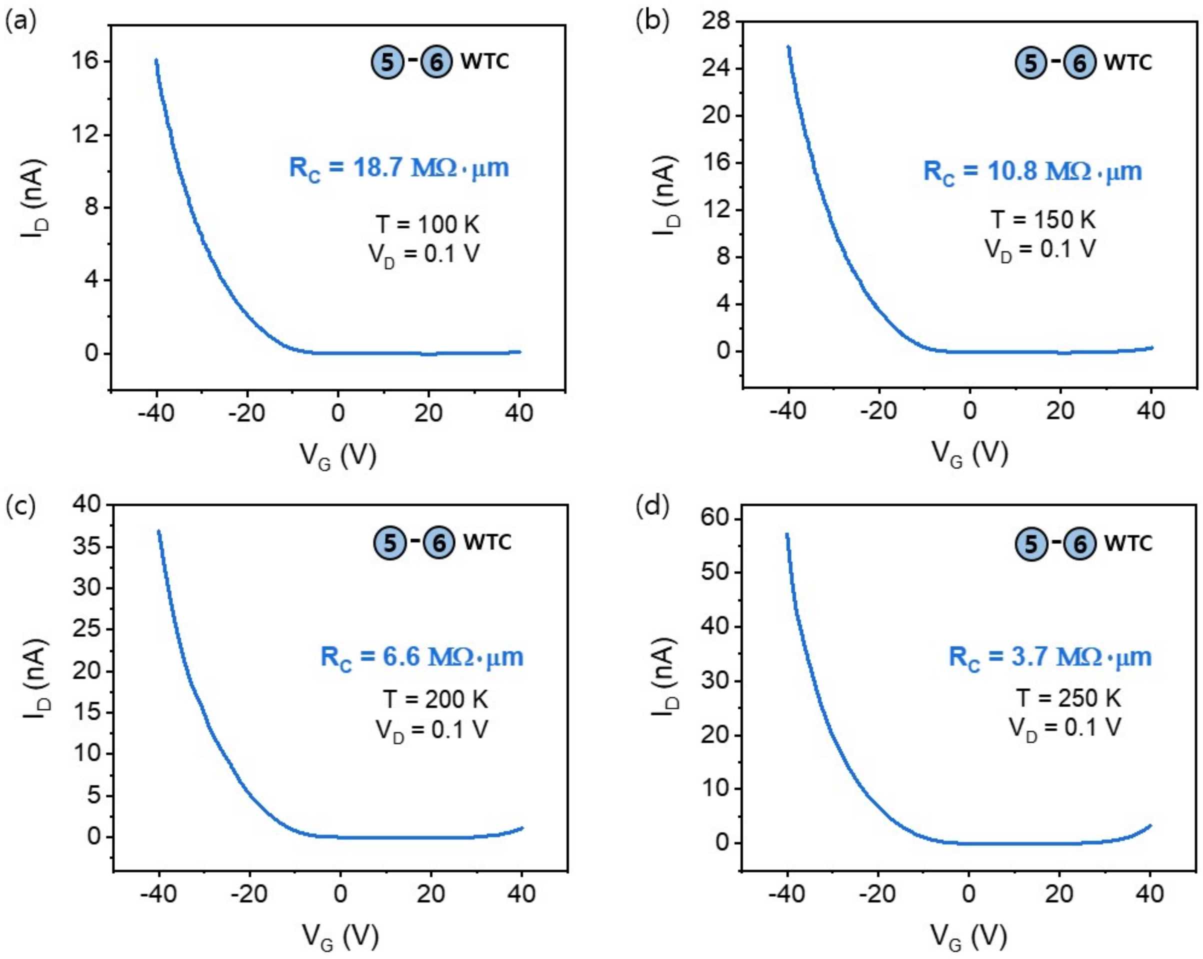

Figure S7. a) I_D-V_G curve of the WTC contact with a contact resistance calculated by the Y-function method at the drain voltage 0.1 V at 100 K, b) 150 K, c) 200 K, and d) 250 K.

References

[1] H. Y. Chang, W. N. Zhu, D. Akinwande, *Appl. Phys. Lett.* **2014**, 104, 113504.

[2] G. Ghibaudo, *Electron. Lett.* **1988**, 24, 543.

[3] Y. Xu, T. Minari, K. Tsukagoshi, J. A. Chroboczek, G. Ghibaudo, *J. Appl. Phys.* **2010**, 107, 114507.
